# 

**Published:** 1887-01-15

**Authors:** 


### The Hospital.

An Institution, Family, and Parochial Journal of Hospitals, Asylums, and all Agencies for the Care of the Sick, Criticism and News.

SATURDAY, JANUARY 15th, 1887.

### THE EVELINA HOSPITAL FOR CHILDREN.

### DEBTOR AND CREDITOR.

A book-keeper walking through Harley Street, and seeing "Dr." on every other house, remarked that it would look a little more regular if there were an occasional "Cr." on the other side of the street.

### THE CHILDREN'S CORNER.

### OUR PASTIMES.

**Children's Quarterly Prize Competition.**

Began .. .. .. .. .. 1st Jan., 1887

Ends .. .. .. .. .. 26th Mar., 1887

PRIZES.

1st .. .. .. .. .. Thirty Shillings

2nd .. .. .. .. .. Twenty „

3rd .. .. .. .. .. Ten „

4th .. .. .. .. .. Five „

will be awarded to the four competitors who shall have sent in, during the thirteen weeks, the largest number of correct answers to our pastimes.

**Special Letter Prize.**

Each competitor is invited to forward a little letter with the answers, addressed to the Puzzle Editor. The letters sent by competitors may be upon any subject they choose, such as their studies, their little pets, their little brothers, sisters or companions, little stories or recollections about the hospitals, or anything else they please. The letters should not be too long, and attention should be paid to the handwriting and the grammar. The Puzzle Editor has decided to award a SPECIAL LETTER PRIZE OF TEN SHILLINGS for the best letters contributed during each Quarterly Competition.

**Third Competition.**

No. 16. REBUS. Who can tell me what is the centre of gravity?

No. 17. GEOGRAPHICAL PUZZLE.

My *first* you'll see, denotes a girl
My *last*, a naughty boy will hurl,
My *whole's*, a town in county Kent,
Now, tell me please, what word is meant?

No. 18. BEHEADALS. Behead a numeral and leave a pronoun, behead an adverb of time and leave a bird, behead a domestic animal and leave a preposition.

No. 20. REBUS. Look through the present number of THE HOSPITAL, and tell me, if you can, which of the letters of the alphabet occurring in it are the most sensible ones.

No. 21. HISTORICAL ENIGMA.

King Charles I laughed,
King Charles I talked
Half an hour after
His head was cut off.

How can the above be made possible without altering or changing the position of a single word?

No. 22. HIDDEN WORDS.

I am a word of letters few,
In fact, I'm formed of only three,
My *first*,—think what all debtors do,
My *next* is more like you than me,
My *third's* like tea—come ,look about,
Try, find the *preposition* out.

**Results of First Competition.**

No. 1. The buried proverb here is "A stitch in time saves nine." All my little friends discovered it.

No. 2. My whole is "skill"; behead, and it becomes "kill"; behead again, and you have "ill."

No. 3. I admit that the similarity between a young lady and a Bill of Exchange is, at first sight, not very striking, hence a little diversity of opinion was only to be expected. Skye suggests that both are marketable commodities, and may be *settled* by *promissory notes*. Tina thinks it must be because the young lady is "presented" when she "comes out"; while Mikado, like a shrewd, business-like little damsel, answers, "Because you must meet your *engagement* or be *sued* upon it! But the answer is this: Marriageable young ladies resemble Bills of Exchange because, you know, they ought always to be *settled* when they arrive at *maturity!*

No. 4. The *lights* in this diagonal puzzle are :—

RHONE

H e N

O

A h N

SAONE

which gives us the two French rivers, the Rhone and the Saone.

No. 5. I am so sorry to say that, through some unfortunate slip, the fourth *light* was omitted from the question; so, in common fairness to my little competitors, I have credited them with No. 5 as right in each case. The omitted *light* was "the opposite of merry," hence the answer stands.

N e W
U n A
R oa R
S a D
E lve S

giving us "Nurse" and "Wards."

No. 6. A Greek or Latin grammar resembles a hospital because it always contains, you know, both *accidence* (accidents) and *cases!*

No. 7. The symbols may be arranged in the following order to fulfil the conditions.

1a 2b 3c 4d

4c 3d 2a 1b

2d 1c 4b 3a

3b 4a 1d 2c

Very nearly all solved this little problem.

All right—None.
Six right—Skye, Florrie, A.C.K., Mikado, Tina, P.T.
Five right—Scrooge, Alsie, Prospect.
Four right—Paul Methuen.
Three right—Filomel, True Blue.
Two right—Isobel. Little Mary, Bismarck.

I need hardly tell you how pleased I am to see such an increase in the circle of my little friends, and I hope that all of them will do what they can to induce any little companions they may have to join with them. My primary purpose in the Children's Corner is, of course, to amuse, and if I succeed in enabling my little guests now and again to pass a pleasant half hour, my object is almost attained. I say *almost*, for I am anxious to make the Corner, to a certain extent, a means of self-improvement. The little letters, for example, will, I hope, serve a double purpose. I want them, as far as possible, to be little models of epistolary correspondence, and I should like the writers to pay particular attention to accuracy of expression and neatness of execution. A nicely written letter is always pleasant to read, but it is surprising to observe how very few persons attain proficiency even in so simple a matter as the art of expressing one's thoughts in words. Now, I want you all to try. Imitate, if you please, the style of the letters you see in our Corner, but always let the composition be *your own*, no matter what the faults may be that you commit. Two or three little letters are to hand this week, a couple of which I print. LITTLE MARY writes:

DEAR MR. EDITOR,—I do so like the CHILDREN'S CORNER. I and Freddy (that is my little brother) are always so anxious for the next number of THE HOSPITAL, and we turn to the puzzles at once and begin upon them. I am going to try very hard to win a prize this time.

Yours faithfully, LITTLE MARY.

BISMARCK writes:—

DEAR EDITOR.—I was going to ask if I might sometimes send you a puzzle or two. Please to let me know in the next number of the Children's Corner.

Believe me, yours truly, BISMARCK.

Yes, I shall be very pleased to receive any good puzzles from my little readers, and when any one happens to appear, the sender will of course be able to answer it and get credit for it. I must thank Miss Rose B. Hawes for the two puzzles which she so kindly sent me last week.

Answers, having the actual name and address (to which a pseudonym may be added for publication), must have "The Children's" Coupon attached (see advertisement pages), and be addressed to the "PUZZLE EDITOR," THE HOSPITAL, Norfolk House, Norfolk Street, Strand, W.C., not later than Thursday, 20th January, 1887. Answers will appear in our issue of the 29th January, 1887.

### "THE HOSPITAL" CHARITY BOX.

WE have received the following letter. Before deciding, we should be glad to know how far the views expressed by Miss Bainbridge are shared by the majority of our readers, and whether they would prefer Acrostics, Poetry, or Historical Competitions, or some other form of amusing puzzle. Answers should reach us by the 18th inst. at latest.—[ED. T. H.]

DEAR SIR,—I am so awfully sorry that you are obliged to discontinue your dissected word competitions in THE HOSPITAL, and I hope you will forgive my writing to you on the subject. The object of the "Charity Box" was so good and the whole thing conducted on such upright principles, that even the unsuccessful would feel some pleasure in knowing their money had gone in a good cause. Would it not be possible to continue, by considerably reducing the prizes; say to ten shillings, seven shillings, and four shillings respectively? The amusement among the competitors would be as great, and I should fancy it increased the circulation of your paper. It was through an advertisement connected with "The Charity Box" that I for one became acquainted with the interesting pages of THE HOSPITAL, and a subscriber, and it may have been the case with many others. Hoping you will pardon the liberty I take, I remain, sir, yours faithfully,

LAURA BAINBRIDGE.

Ashton House, Jersey.
Jan. 3rd.

### NOTICE TO CORRESPONDENTS.

All correspondence must be written on one side of the paper only.
We cannot undertake to return MSS. not used.

**Communications**, whether intended for insertion or for private information, must be authenticated by the names and addresses of th writers, not necessarily for publication.

**Local papers** containing reports or news paragraphs **should be marked.**

### THE IN- AND OUT-PATIENTS' HOSPITAL DIARY.

This Diary is so arranged as to make some portion of it appear every week, and the whole in each monthly part. It has been very difficult to compile, and corrections will at all times be gratefully received. It has been suggested that similar information about the larger Provincial and Scotch Hospitals would be useful to many readers. We should be glad to hear if this is felt to be the case.

### GENERAL HOSPITALS.—*continued.*

| Hospitals and Terms of Admission. | Physicians, Surgeons, and Medical Officers, with Days and Hours of Attendance. |
|---|---|
| St. Mary's Hospital, Cambridge Place, Paddington, W. Sec., Mr. Pietro Michelli. In-patients are admitted by letter of recommendation. Urgent cases and accidents free at all times. Out-patients require a letter. Electrical treatment Tu. and F. 2 | *In-Physicians*, Sir Edward Sieveking, Tu. F. 1.45; Drs. Broadbent, M. Th. 1.45; Cheadle, W. 1.45, S. 9.30. *In-Surgeons*, Messrs. Norton, M. Th. 1.45; Owen, Tu. F. 1.45; H. Page, W. S. 1.45. *Out-Physicians*, Drs. D. B. Lees, W. S.; S. Phillips, M. Th; R. Macguire, Tu. F. Hour, 1.30. *Out-Surgeons*, Messrs. W. Pye, M. Th.; A. J. Pepper, Tu. F.; A. Q. Silcock, W. S. Hour, 1.30 |
| St. Thomas's Hospital, Westminster Bridge Road, S.E. Steward, Mr. F. Walker. Accidents and emergencies free at all times. Other cases by Governor's letter, but, where unobtainable, application should be made to the Steward by letter, or personally between 10 and 4. Vaccination on W. at 11.30 | *In-Physicians*, Drs. Bristowe, Tu. F.; Stone, M. Th.; Ord, M. Th.; J. Harley, Tu. F.; Gervis, Tu. F. Hour, 2. *In-Surgeons*, Messrs. Sydney Jones, Tu. F.; Croft, M. Th.; Sir W. MacCormac, M. Th. Hour, 2. *Out-Physicians*, Drs. Payne, Tu. F; Sharkey, M. Th.; Gulliver, W. S. Hour, 1.30. *Out-Surgeons*, Messrs. Clutton, Tu. F.; Anderson, W. S.; Pitts, M. Tu. Hour, 1.30 |
| Tottenham Training Hospital, Tottenham, N. Director, Dr. Laseron. Free. Accidents and emergencies admitted at all times | *In-Physician*. Dr. Rasch, Tu. F. 3 to 5. *In-Surgeons*, Drs. Lichtenberg, M. Th. 3 to 5; E. H. May, M. Th. 3 to 5. *Out-Surgeon*, Dr. Lloyd Smith, M. W. 11 to 2; *Men* only, W. 7 to 8 p.m. |
| University College Hospital (or North London), Gower Street, W.C. Sec., Mr. N. H. Nixon. Accidents and emergencies free. All other cases by Governor's letter of recommendation | *In-Physicians*, Drs. W. Fox, Tu. Th. 2, S. 9.30; Ringer, M. W. F. 1.30; Bastian, M. 2.30, W. 10, F. 2.30; F. Roberts, Tu. 1.15, Th. 1.30, S. 9.30. *In-Surgeons*, Messrs. B. Hill, Tu. 2.15, W. 2, F. 2.15; C. Heath, M. W. F. 2; M. Beck, Tu. Th. S. 2. *Out-Physicians*, Drs. Gowers, W. S.; Poore, Tu. F.; Barlow, M. Th. Hour, 1.30. *Out-Surgeons*, Messrs. Barker, M. Th.; Godlee, Tu. F.; Horsley, W. S. Hour, 1.30 |
| West London Hospital, Hammersmith Road, W. Sec. and Supt., Mr. R. J. Gilbert. Accidents and emergencies free at all times. Other cases require a letter of recommendation. New cases must attend at 1.30 | *In-Physicians*, Drs. G. Rogers, D. W. Hood, F. Drewitt. *In-Surgeons*, Messrs. C. Keetley, F. Edwards, W. B. Clarke. *Out-Physicians*, Drs. Herringham, M. Th.; Ball, Tu. F.; S. Taylor, W. S. Hour, 2. *Out-Surgeons*, Messrs. Ballance, Tu. F.; Weiss, M. Th.; Wainewright, W. S. Hour, 2 |
| Westminster Hospital, Broad Sanctuary, S.W. Sec., Mr. S. M. Quennell. Accidents and urgent cases free at all times. Other cases require a letter of recommendation | *In-Physicians*, Drs. Sturges, M. Th. S.; Allchin, M. Tu. F.; Donkin, Tu. W. S. Hour, 1.30. *In-Surgeons*, Messrs. Cowell, M. Tu. Th.; Davy, M. Tu. F.; Macnamara, M. W. S. Hour, 1.30. *Out-Physicians*, Drs. De H. Hall, M. Th.; A. H. Bennett, Tu. F.; Murrell, W. S. Hour, 1.30. *Out-Surgeons*, Messrs. Cooke, M. Th.; Bond, Tu. F.; Barrow, W. S. Hour, 1.30 |

### II.—SPECIAL HOSPITALS.

### CANCER.

| Hospitals and Terms of Admission. | Physicians, Surgeons, and Medical Officers, with Days and Hours of Attendance. |
|---|---|
| Cancer Hospital, Brompton, S.W. Sec., Mr. W. H. Hughes. Entirely free to both in- and out-patients | *In- and Out-Surgeons*, Drs. A. Marsden, W.; H. Snow, M.; F. A. Purcell, F.; Messrs. F. B. Jessett, W.; C. Stonham, Th.; C. Jennings, Tu.; W. H. Elam, S. Hour, 2 |
| London Hospital, Whitechapel Road, E. | Daily, 1.30. *See* General Hospitals |
| Middlesex Hospital, Berners Street, W. Admission free. | *In-Surgeons*, Messrs. Hulke, Lawson, and Morris. *Out-Surgeon*, Mr. Morris, Th. 1.30 |
| St. Saviour's Hospital, Osnaburg Street, N.W. Sec., Mr. E. Poitiers. By Governors' letters | *In- and Out-Physician*, Dr. A. Kennedy, 10.30 and 4 daily |

### CHILDREN'S DISEASES.

| Hospitals and Terms of Admission. | Physicians, Surgeons, and Medical Officers, with Days and Hours of Attendance. |
|---|---|
| Alexandra Hospital for Children, 18, Queen Sq., W.C. Sec., Mrs. Marsh. For hip disease | *Physician*, Dr. Steavenson. *Surgeons*, Messrs. H. Marsh Tu. F.; A. Bowlby, M. Th. Hour 4.30 (No out-patients) |
| All Saints' Children's Hospital, 4, Margaret Street, W. For chronic joint diseases. | *Surgeon*, Mr. N. Smith. (No out-patients) |
| Belgrave Hosp. for Children, 77 & 79, Gloucester Street, Pimlico. Hon. Secs., Capt. Stopford, and Rev. J. Storrs. By letters; accidents free | *In- and Out-Physicians*, Drs. W. Hope and W. Ewart, M. Th. 9. *In- and Out-Surgeons*, Messrs. C. T. Dent and Margerison, Tu. F. 9 |
| Charing Cross Hospital, West Strand, W.C. | *Out-Physician*, Dr. Nursby, W. S. 1.30 |
| Cheyne Hospital for Sick and Incurable Children, 46, Cheyne Walk, Chelsea, S.W. Sec., Miss D. Evans | *Physician*, Dr. G. V. Poore. *Surgeons*, Messrs. T. P. Bartlett and J. Macready attend alternate days in afternoons. (No out-patients) |
| East London Hospital for Children and Dispensary for Women, Glamis Road, Shadwell, E. Sec., Mr. Ashton Warner. By Governor's letter. Urgent cases and accidents are admitted free | *In-Physicians*, Drs. Donkin, M. Th.; Eustace Smith, T. F.; Crocker, F. *In-Surgeons*, Messrs. Calder, W. F.; Parker, W. F. *Out-Physicians*, Drs. Crocker, M.; Williams, Tu. Th.; Coutts, W. F. Hours, 1 and 3. *Out-Surgeon*, Mr. Dunn, Tu. F. 1, 3 |
| Evelina Hospital for Sick Children, Southwark Bridge Road, S.E. Sec., Mr. T. S. Chapman. Free, but Governor's letter takes precedence. In-patients (boys) between 2 and 10, (girls) between 2 and 12 | *In-Physicians*, Drs. F. Taylor and J. Goodhart. *In-Surgeons*, Messrs. H. G. Howse and R. C. Lucas. *Out-Physicians*, Drs. N. Tirard, M. Th. 9; F. Willcocks, Tu. F. 9. *Out-Surgeons*, Messrs. C. J. Symonds, S. 9; G. H. Makins, W. 9 |
| German Hospital, Dalston Lane, Dalston, E. | *In-Physician*, Dr. Rasch, M. Th. 9 |
| Great Northern Central Hospital, Caledonian Rd. N. | *Physicians*, Drs. Murray and Barnes, W. 2.30 |
| Grosvenor Hospital for Women and Children, Vincent Sq., S.W. Hon. Secs., Mr. A. J. Ram and Miss Day. By letter or payment | *In- and Out-Physicians*, Drs. R. Gibbons and Steavenson, Tu. W. Th. S. 2. *In- and Out-Surgeons*, Messrs. Smythe and Parker, Tu. W. Th. S. 2 |
| Hospital for Sick Children, 48 and 49, Great Ormond St., W.C. Sec., Mr. S. Whitford. By Governors' letters. If without letter, the case of the applicant is investigated. In-patients between 2 and 12; Out-patients under 12 | *In-Physicians*, Drs. W. Cheadle, Tu. F.; Sturges, W. S.; Barlow, M. Th. Hours, 9 to 11. *In-Surgeons*, Messrs. H. Marsh, W. S.; E. Owen, W. S. Hours, 9 to 11. *Out-Physicians*, Drs. R. J. Lee and Abercrombie, M. Th.; Lubbock and Money, Tu. F. Hours, 8.30 to 10. *Out-Surgeons*, Messrs. Morgan and Pitts, W. S. 8.30 to 10 |
| King's College Hospital, Portugal Street, W.C. | *In- and Out-Physician*, Dr. T. C. Hayes, W. F. 1 |
| Middlesex Hospital, Berners Street, W. Free. Children under 3. | *Out-Physician*, Dr. Duncan, W. S. 1.30 |
| North Eastern Hospital for Children, Hackney Road, E. Sec., Mr. A. Nixon. By free ticket or small payment. | *In- and Out-Physicians*, Drs. W. Cayley, F. Turner, C. Semple, and W. Pasteur, M. W. Th. S. 1.30. *In- and Out-Surgeons*, Messrs. Waren Tay and Pollard, M. 1.30, F. 9 |
| Paddington Green Children's Hosp., Paddington Green. Sec., Mr. W. H. Pearce. Admission free; boys under 12; girls under 14 | *In- and Out-Physicians*, Drs. T. Lauder Brunton, Ogilvie, Tu. F.; G. Laycock, M. Th.; S. Phillips, W. S. Hour, 9.15. *In- and Out-Surgeons*, Messrs. W. W. Cheyne, M., S. Boyd, F. Hour, 9.15 |
| Royal Hospital for Children and Women, Waterloo Bridge Road, S.E. Sec., Mr. R. G. Kestin. Out-Patients pay 1*d*. on each attendance. Boys not admissible, as out-patients, over 12, or, as in-patients, over 8 | *In-Physicians*, Drs. Park, M. Th.; Gulliver, Tu. F.; Price, W. S. Hour, 2. *In-Surgeon*, Mr. W. H. A. Jacobson, M. Th. Hour 2. *Out-Physicians*, Drs. Park, M. Th.; Dakin, W. F.; Hadden, Tu. S. Hour, 2. *Out-Surgeon*, Mr. E. O. Day, W. 2 |
| St. Mary's Hospital, Cambridge Place, Paddington. | *Out-Physician*, Dr. M. Handfield Jones, M. Th. 1.30 |
| St. Thomas's Hospital, Westminster Bridge Road, S.E | *Out-Physician*, Dr. Cory, W. S. 1.30 |

### CHILDREN'S DISEASES.—*continued.*

| Hospitals and Terms of Admission. | Physicians, Surgeons, and Medical Officers, with Days and Hours of Attendance. |
|---|---|
| Samaritan Free Hospital for Women and Children, Lower Seymour Street, W.; Branch, 1, Dorset St., W. Sec., Mr. G. Scudamore. In-patients must be between 2 and 12. Out-patients Department (free) is at Edward's Mews, Duke Street, W. | *In-Physician,* Dr. W. H. Day. Daily, 2 *In-Surgeon,* Mr. W. A. Meredith. Daily, 2 *Out-Physicians,* Drs. M. Prickett and Amand Routh, W. S. 1.30 *Out-Surgeons,* Messrs. W. A. Meredith and J. D. Malcolm, M. Th.; A. H. G. Doran and W. S. A. Griffith, Tu. F., 1.30 |
| Victoria Hosp. for Children, Queen's Road, Chelsea, S.W. Sec., Capt. W. C. Blount, R.N. Age of boys, 2 to 12 years; age of girls, 2 to 16. Admission for in-patients by subscriber's letter. Accidents and urgent cases free at all times | *In-Physicians,* Drs. J. Evans, M. Th. 2; Ridge-Jones, Tu. F. 2 *In-Surgeons,* Messrs. Pick, Tu. F. 9.15; Clutton, M. Th. 4.30 *Out-Physicians,* Drs. A. Venn, W. S. 9.30; C. Fox, M. Th. 1 30; F. D. Drewitt, Tu. F. 9; J. Philpot, M. Th. 9 *Out-Surgeons,* Messrs. F. Churchill, Tu. F. 10; W. Pye, M. Th. 9.30 |
| West London Hospital, Hammersmith rd, W. | Daily, 2. *See* General Hospitals |

### CONSUMPTION AND DISEASES OF THE CHEST.

| | |
|---|---|
| City of London Hospital for Diseases of the Chest, Victoria Park, E. Sec., Mr. T. Storrar Smith By subscriber's letter available for 6 weeks. | *In-Physicians,* Drs. J. Thorowgood, S.; E. Smith, M.; J. Fothergill, W.; S. West, F.; G. A. Heron, W.; V. D. Harris, Tu. Hour, 2 *Out-Physicians,* Drs. V. D. Harris, Tu.; J. A. Ormerod, Th.; E. C. Beale, Tu. F.; B. Fenwick, W. S.; A. Money, W. S.; L. E. Shaw, M. Th. Hour, 2 |
| Hospital for Consumption, Fulham road, Brompton, S.W. Sec., Mr. H. Dobbin Admission by letter of recommendation, but, where unobtainable, application may be made to the Secretary at the Hospital. | *In-Physicians,* Drs. E. S. Thompson, M. Th.; C. T. Williams, M. Th.; R. D. Powell, Tu. F.; J. Tatham, W. S.; R. E. Thompson, W. S.; F. T. Roberts, Tu. F. Hours, 2 to 4 *In-Surgeon,* Mr. R. J. Godlee, F. *Out-Physicians,* Drs. T. H. Green, J. M. Bruce, M. Th.; J. K. Fowler, W. S.; P. Kidd and C. Y. Biss, Tu. F.; T. D. Acland, W. S. Hour, 12 |
| North London Hospital for Consumption and Diseases of the Chest, Mount Vernon, Hampstead, N. Sec., Mr. L. F. Hill Admission by subscriber's letter available for 6 weeks. | *In-Physicians,* Drs. A. Evershed, Tu.; B. O'Connor, F.; D. Pidcock, F. Hour, 10.30 *Out-Physicians,* Drs. B. O'Connor, D. Pidcock, J. E. Squire. Daily, 2 (attend at Out-patients' Branch, 216, Tottenham Court Road, W.) |
| Royal Hospital for Diseases of the Chest, 231, City Rd., E.C. Sec., Mr. John J. Austin By Governor's letter, available for six weeks | *In-Physicians,* Drs. Hensley, W.; Gilbart-Smith, M.; Finlay, Th. *In-Surgeon,* Mr. A. Pearce-Gould, M. Tu. W. Th. F. 2, S. 9 *Out-Physicians,* Drs. White, Tu. F.; Pringle, M. Th.; O. Browne, W. S.; A. Davies, M. F.; H. Habershon, W. S.; E. Stewart, Tu. Th. Hour, 1; S. 9 |
| Royal National Hosp. for Consumption and Diseases of the Chest, Ventnor. Offices, 34, Craven St., Strand, W.C. Sec., Mr. E. Morgan Admits only incipient cases by Governor's letter. | *In-and Out-Physicians,* Drs. J. G. Sinclair Coghill, and R. Robertson attend daily (at Ventnor) on out-patients at 11; on in-patients at noon *In- and Out-Surgeons,* Messrs. Whitehead, Williamson |

### EAR CASES.

| | |
|---|---|
| Central London Throat and Ear Hosp., Gray's Inn Rd., W.C. Sec., Mr. R. Kershaw Free, but when able, a small payment is expected | *In- and Out-Surgeons,* Mr. Lennox Browne, M, Th. 2.30; Dr. Orwin, W. S. 2.30; Dr. Grant, Tu. 5, Th. 2.30; Messrs. P. S. Jakins, M. W. 2.30; F. 5; T. Carmalt Jones, M. Th. S. 2.30 |
| Charing Cross Hospital, West Strand, W.C. | *In- and Out-Surgeon,* Mr. Cantlie, F. 9 |
| Great Northern Central Hospital, Caledonian Rd, N. | *In- and Out-Surgeon,* Mr. A. E. Cumberbatch, W. 9 |
| Guy's Hospital, St. Thomas's Street, S.E. | *In- and Out-Surgeon,* Mr. Purves, Tu. F. 12.30 |
| Hospital for Diseases of the Throat, Golden Square, W. Sec., Mr. W. T. Sharp | *Physicians,* Drs. Wolfenden, Tu. F. 2.30; Macdonald, Tu. F. 6.30; Bond, W. S. 2.30 *Surgeon,* Mr. T. M. Hovell, M. Th. 2.30 |

### EAR CASES—*Continued.*

| Hospitals and Terms of Admission. | Physicians, Surgeons, and Medical Officers, with Days and Hours of Attendance. |
|---|---|
| King's College Hospital, Portugal Street, W.C. | *In- and Out- Surgeon,* Mr. Pritchard Th. 2.30 |
| London Hospital, Whitechapel Road, E. | *In- and Out-Surgeons,* Dr. Woakes, Mr. T. M. Hovell, S. 9 |
| Middlesex Hospital, Berners Street, W. Free. | *Out-Surgeon,* Mr. Hensman, Tu. 9 |
| National Hosp. for the Paralysed, etc., Queen Sq., W.C. | *In- and Out-Surgeon,* Mr. A. E. Cumberbatch, W. 2 |
| North West London Hospital, 18, Kentish Town Rd. | *Out-Surgeon,* Mr. Collier, M. 1.30 |
| St. Bartholomew's Hospital, Smithfield, E.C. | *Surgeon,* Mr. Cumberbatch, Tu. F. 2 |
| St. George's Hospital, Hyde Park Corner, S.W. | *In- and Out-Surgeon,* Sir W. B. Dalby, Tu. 1.30 |
| St. Mary's Hospital, Cambridge Place, Paddington, W. | *In- and Out-Surgeon,* Mr. G. P. Field, W. S. 9.30 |
| St. Thomas's Hospital, Westminster Bridge Road, S.E. | *Out-Surgeon,* Mr. Clutton, M. 1.30 |
| University College Hospital, Gower Street, W.C. | *In- and Out-Surgeon,* Mr. Barker, Th. 9 |
| Westminster Hospital, Broad Sanctuary, S.W. | *In- and Out-Surgeon,* Mr. Barrow, Tu. F. 9 |

### EYE DISEASES.

| | |
|---|---|
| Central London Ophthalmic Hosp., 238A, Gray's Inn Rd., W.C. Sec., Mr. G. H. Leah Admission free | *Surgeons,* Messrs. T. Archer, G. Abbott, W. Charnley, W. Jessop, E. Clarke, J. R. Kemp and Granville Barker (House Surgeon). Daily, 1 |
| Evelina Hosp. for Sick Children, Southwark Bridge Rd. | *Surgeon,* Dr. W. Brailey, Th. 1 |
| Great Northern Central Hospital, Caledonian Rd., N. | *Surgeon,* Mr. Hutchinson, Jnr., Tu. F. 9.30 |
| Guy's Hospital, St. Thomas's Street, S.E. | *Surgeons,* Mr. Higgens, Dr. Brailey, Tu. F. 12.30 |
| Hospital for Sick Children, 49, Gt. Ormond Street, W.C. | *Surgeon,* Mr. R. Marcus Gunn, Tu. 2 |
| King's College Hospital, Portugal Street, W.C. | *Surgeon,* Mr. McHardy, M. W. Th. 1.30 |
| London Hospital, Whitechapel Road, E. | *Surgeons,* Messrs. Waren Tay, S. 9; Eve, Tu. 9 |
| Middlesex Hospital, Berners Street, W. | *Surgeon,* Mr. Lang, Tu. F. 9 |
| National Hosp. for the Paralysed, etc. Queen Sq., W.C. | *Surgeons,* Messrs. R. Brudenell Carter, F. 4; R. Marcus Gunn, Tu. 4 |
| North-West London Hosp. 18, Kentish Town Rd., N.W. | *Surgeon,* Dr. W. Collins, F. 3.30 |
| Paddington Green Children's Hosp., Paddington Grn. | *Surgeon,* Mr. W. H. H. Jessop, Th. 9 |
| Royal Free Hospital, Gray's Inn Road, W.C. | *Surgeon,* Mr. Mackinlay, M. F. 9 |
| Royal London Ophthalmic Hospital, Blomfield Street, Moorfields, E.C. Sec., Mr. R. J. Newstead Admission free to the poor. | *Surgeons,* Messrs. J. W. Hulke, W. S.; G. Lawson, Tu. F.; J. Couper, Tu. F.; W. Tay, M. Th.; J. Tweedy, Tu. F.; E. Nettleship, W. S.; R. M. Gunn, W. S.; Lang, M. Th. Hours, 8 to 10 |
| Royal South London Ophthalmic Hosp., 6, St. George's Circus, S.E. Sec., Mr. C. Comyn Free to the necessitous poor; or by payment | *Surgeons,* Messrs. Watson, McHardy. Daily, 2 |
| Royal Westminster Ophthalmic Hospital, King William Street, W.C. Sec., Mr. T. Beattie-Campbell Poor and accidents free | *In- and Out-Surgeons,* Messrs. Power. M. F.; Frost, M. Th.; Rouse, Tu. S.; Hartridge, Tu. F.; Macnamara, M. Th.; Cowell, W. S.; Juler, W. S. Hour, 1 |
| St. Bartholomew's Hospital, Smithfield, E.C. | *Surgeons,* Messrs. Power, Th. 2; Vernon, Tu. S. 2.30 |
| St. George's Hospital, Hyde Park Corner, S.W. | *Surgeons,* Messrs. B. Carter, Frost, M. Th. 2; F. 1.15; W. S. 2 |
| St. Mary's Hospital, Cambridge Place, Paddington. W. | *Surgeons,* Messrs. A. Critchett, H. Juler, Tu. F. 9.30 |
| St. Thomas's Hospital, Westminster Bridge Road, S.E. | *Surgeons,* Messrs. Nettleship, Tu. Th. F. 1.30; Lawford, M. W. 1.30 |
| University College Hospital, Gower Street, W.C. | *Surgeon,* Mr. Tweedy, M. Tu. Th. F. 2 |
| West London Hospital, Hammersmith Road, W. | *Surgeons,* Messrs. B. Vernon, H. Dunn Tu. Th. S. 2 |
| Westminster Hospital, Broad Sanctuary, S.W. | *Surgeon,* Mr. Cowell, M. Th. 2 |
| Western Ophthalmic Hosp. 153 and 155, Marylebone Rd., W. Sec., Mr. G. E. Manton. By letter or small payments; free to poor | *Surgeons,* Drs. Miller, M.; Collins, F.; Wheatly, M. Th.; Messrs. Carmalt Jones, W.; Buxton, Tu. F.; Nunn, W. S. Hour, 2 |

